# Cognitive Disposition to Wine Consumption: How the Brain Is Wired to Select the Perfect Bottle With a Novel Musical Twist

**DOI:** 10.3389/fnins.2019.01157

**Published:** 2019-11-22

**Authors:** Gabriella Soós, József Csernák, László Lakatos, Zsolt Zsófi, András Palotás

**Affiliations:** ^1^Eszterházy Károly University, Eger, Hungary; ^2^Asklepios-Med (Private Medical Practice and Research Center), Szeged, Hungary; ^3^Kazan Federal University, Kazan, Russia

**Keywords:** decision neuro-science, multi-sensory environment, Mozart-effect, music, neuro-marketing, perception, taste, wine

## Abstract

Taste is not a veridical perception: it is modifiable by cognitive and affective processes, as well as by expectations. Even though molecular composition determines the savor, various other factors such as external characteristics and basic assumptions have a sway over perceived pleasantness of food and drink. The rituals associated with wine tastings especially underscore the importance of these subjective ramifications. While auditory stimuli are known to influence drinking experience, the impact of melody on the product itself and on corollary consumer preference is unknown. As routine judgments are also influenced by informational cues, here we evaluated whether description of a unique technological innovation (i.e., serenaded grapes and barrels) as cogent suggestion of quality improves willingness to buy. This project unveils that the belief about music-fed wines, which might be construed as a motivational bias, can alter purchasing decisions; however, further neuro-marketing studies are warranted with this respect.

## Introduction

While cognitive regulation of decision-making is yet to be fully understood, it is widely accepted that multi-sensory environment has a significant impact on wine-drinking experience. The setting/scenery, hue of the ambient lighting, or background music, just to name a few, can effectively shape how people rate the taste of a drink (Pangborn et al., 1963; Morrot et al., 2001; Oberfeld et al., 2009; Spence and Deroy, 2013; Wang and Spence, 2017; Hauck and Hecht, 2019). In addition to such sensory modulations, consumer behavior is also influenced by informational cues. Perceptions of excellence and superiority are known to be positively correlated with escalated market value (Rao and Monroe, 1989). Equally, signals of quality, such as high vs. low price tag of the same wine, may alter ratings of taste pleasantness (Plassmann et al., 2008; Schmidt et al., 2017). Apparently, the extrinsic characteristics of a bottle, such as packaging, price, and brand, are certain to be encountered in this money-driven world; however, the intrinsic wine attributes other than sensory-hedonic values are less emphasized.

Whether it is regarded as a means for enjoyment or self-expression, a communication tool or an important adjunct to religious and social rituals, a source of entertainment or revenue, a trigger for inspiration, or facilitator of relaxation and healing, music has played key roles in every generation in all cultures around the globe. It embraces the whole of humanity and, arguably, even has the power to change the world. There is further evidence that classical music – particularly the works of Wolfgang Amadeus Mozart – may enhance brain activity, cognition, and motor skills, and its beneficial effects have also been implicated in cardiovascular disorders, cancer pain, epilepsy, depression, and dementia (Rauscher et al., 1993; Pauwels et al., 2014; Verrusio et al., 2015; Demarin et al., 2016). Piling data implicate that what we hear can influence what we taste, and there is a remarkably strong relationship between music and wine (North, 2012; Hauck and Hecht, 2019). While auditory stimuli have been shown to modify the drinking experience, the effect of melody on the beverages themselves is essentially unknown. This mainly boils down to the belief that only humans, other vertebrates, and some arthropods possess the sense of hearing. In spite of this, plants are equally receptive to changes in light, and are thought to respond to chemicals, touch, and vibration. More than that, their ability to “hear sounds” is not just anecdotal either. Indeed, it is well-established that sonic waves influence plant growth, promote ATPase activity, and have impact on genome-wide transcription. They are also believed to significantly increase the yield of sweet pepper, cucumber, tomato, lettuce, spinach, cotton, rice, and wheat (Ma et al., 2001; Braam, 2005; Baldwin et al., 2006; De Luca and Vallejo-Marín, 2013; Monshausen and Haswell, 2013; Appel and Cocroft, 2014; Hassanien et al., 2014; Kim et al., 2018). Sonic vibrations could be picked up by mechano-receptors, which are common in these vegetables (Monshausen and Gilroy, 2009). In fact, plants can rapidly respond to sound waves. Exposing flowers to the noise of pollinators such as flying bees or synthetic sound signals of comparable frequency triggers sweeter nectar production within just a few minutes (Veits et al., 2019).

Utilizing a novel approach based on this evidence, AM-Tokaj winery^1^ – located in Hungary’s world-famous Tokaj wine-producing region – plays classical music to its grapevines and barrels every day of the year. The scientific knowledge on the subject at hand is almost non-existent; however, research has recently found that ultra-sound, while not audible to humans, improves the extraction of characteristic components from grapes and promotes the aging of wines (Delgado-González et al., 2017; Romero-Díez et al., 2019). Indeed, we have noted that our plantations respond positively to classical melodies: conveying a heavenly concerto for the palate, the Mozart effect translates into truly congenial aromas and flavors, and has already increased the in-house ratings of AM-Tokaj’s unique products.

Context alters value, which can be increased among many others by quality, esthetics, rarity, or special production technology (Danner et al., 2017; Jiang et al., 2017; Thach et al., 2018). A totally unique, unprecedented method of viti-culture, however, might also trigger aversion of some customers; nevertheless, their purchasing decisions can be influenced by various neuro-marketing tools (Lee et al., 2007; Breiter et al., 2015). Even though the anterior cingulate, orbito-frontal, ventro-medial, and dorso-lateral pre-frontal cortex, as well as sub-cortical regions (amygdala and ventral striatum) have been implicated in decision-making processes (Gold and Shadlen, 2007; Rushworth and Behrens, 2008), very little is known about the neural mechanisms through which emotions and expectations affect decisions made by individuals. Notwithstanding, the purpose of this study was to evaluate how marketing actions can shape willingness to buy. At the intersection of brain research, psychology, economics, and marketing, here we employed an innovative decision neuro-science application to assess cue-based expectancy and to monitor consumer attitude toward excitingly innovative “music-fed” wines with high added value.

## Materials and Methods

A quantitative research was conducted between December 2018 and April 2019. A freely accessible online survey was performed and distributed through various e-forums and social media in the Hungarian language during this period.

The questionnaire contained three main sections: the first segment largely focused on drinking patterns and musical preferences of the individuals. The next part provided an in-depth exploration of the respondents’ beliefs about the potential effects of music on wine, and the putative relationship between music-fed beverages and consumer behavior, including price sensitivity. Finally, demographic data of participants were also collated such as age, gender, level of education, monthly income, marital status, family size, etc.

Using SPSS-23 software, various analytical methods (mainly descriptive statistics and relationship analysis) were applied to make statistical calculations.

A total of 139 appraisable responses were gathered and parsed. While the initial sample was not representative, the data were stratified and weighted by sex based on the readily available information of the Hungarian central statistical office (*Központi statisztikai hivatal*, Budapest), thereby maintaining representativeness by gender.

## Results

### Demographics

The average age of the respondents was 39.5 years. The distribution was normal with a moderate right skew. The range is from 19 to 66 (i.e., 47) years. As wine is an alcoholic beverage, all of the participants were at least 18 years of age, in keeping with Hungarian regulations. The majority in the sample were married, held some form of a higher education degree, and hailed from urban areas (Table 1).

**TABLE 1:** Demographics of participants.

**Variables**		**% (*n* = 139)**
Age (years)	18–30	28.1
	31–40	19.4
	41–50	37.4
	51–65	13.7
	>65	1.4
Qualification	Elementary school	2.2
	High school	31.7
	College/university	59.1
	Post-graduate degree	7.1
Place of residence	Village	21.3
	Town	36.0
	Major city	31.9
	Capital (Budapest)	10.8
Marital status	Single	22.4
	Partnership	32.1
	Married	40.6
	Divorced	4.3
	Widowed	0.6
Net income/person/month (in Hungarian forints, HUF)^∗^	<100,000 (<€308, $345, £278)	12.2
	101–200 k (€309–615, $346–690, £279–555)	40.5
	201–300 k (€616–923, $691–1.034, £556–833)	20.0
	301–400 k (€924–1231, $1035–1380, £834–1111)	14.3
	>400 k (>€1231, $1380, £1111)	12.9

*^∗^Self-reported figures, current exchange rate: 1 EUR = 325 HUF, 1 USD = 290 HUF, 1 GBP = 360 HUF.*

### Musical Orientation

**FIGURE 1:**
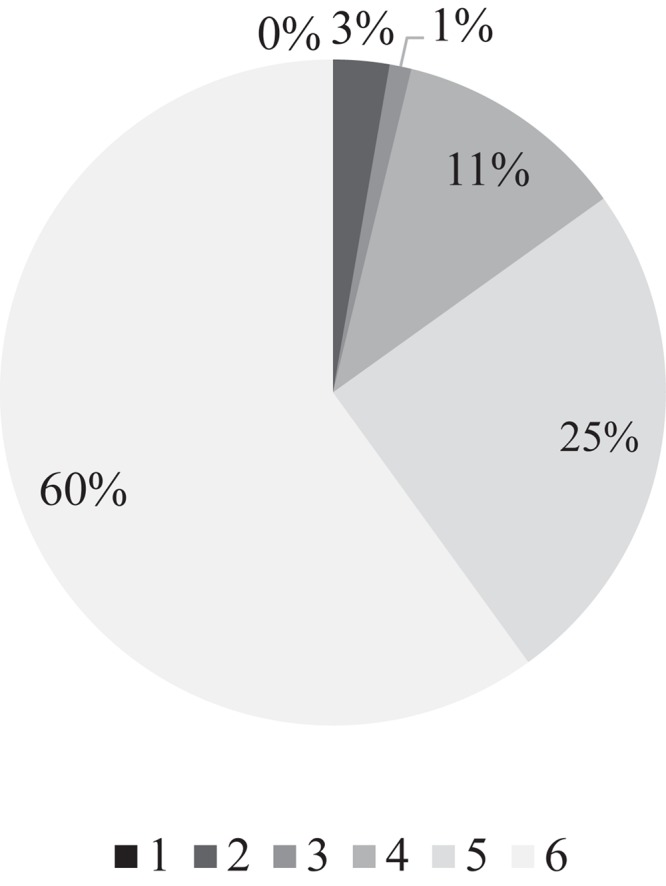
Popularity of music among the respondents. Participants were asked how much they liked music on a scale from 1 to 6, where 1 (black) denoted that they did not like music at all, and 6 (white) suggested that they enjoyed music very much.

On a scale from 1 to 6, participants were asked how much they liked listening to music. All of them seemed to enjoy it to at least some extent (i.e., nobody disfavored melody), and over half of the respondents (60%) viewed music as an important role-player in their lives (Figure 1). More than half of the population (57.6%) cherish various tunes every day, and on average, they listen to music 5–6 days a week (mean = 5.85 days).

**FIGURE 2:**
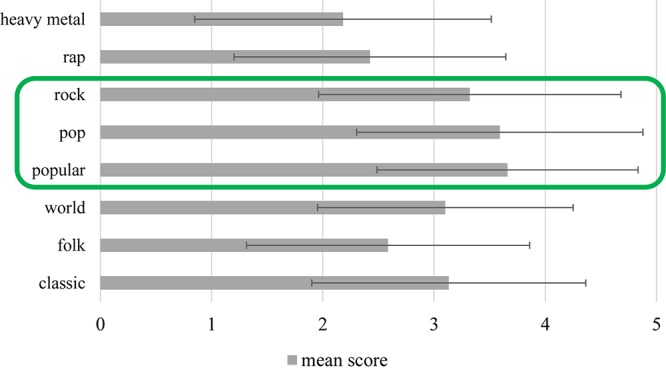
Music preferences of participants. This graph delineates the likes (score: 5) and dislikes (score: 1) of respondents with respect to various music styles. Participants enjoyed listening to popular, pop, and rock music the most.

The personal preference of various music categories was also evaluated using a five-point grading scale, where 1 denoted a strong dislike for and 5 represented a total devotion to a particular style (Figure 2). Popular, pop, and rock music topped the poll, and classical melodies constituted the fourth most widely preferred genres. Coming bottom of the list was heavy metal. A quarter of the respondents play some form of instrument.

**FIGURE 3:**
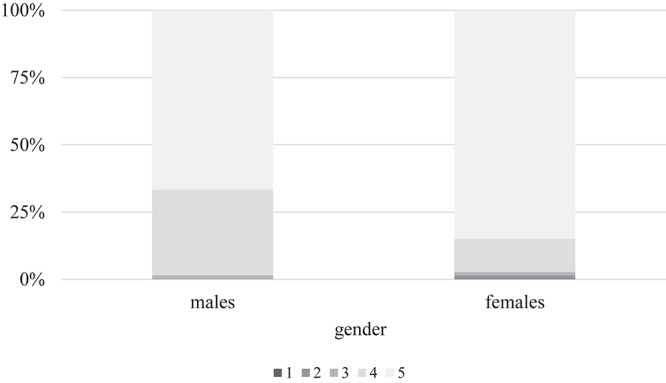
Presumed effect of music on humans as felt by males vs. females. On a five-point grading scale, where 1 represents strong negative influence, 3 denotes no effect, and 5 means strong positive force, participants were asked how they felt music might impact the human body. While the majority believes in a favorable interaction, this figure demonstrates that apparently females trust much more in the beneficial effects of music on humans than their male counterparts.

Participants generally agree with the positive effects of music on the human body: the average score on the five-point scale was 4.74 with starkly right-skewed distribution, suggesting that people understand the beneficial impact of sound compositions on mankind. However, there is a significant gender difference as females largely believe in the positive effects of melody more than males (Φ = 0.248, α = 0.036) (Figure 3).

### Smoking and Other Forms of Addiction

Because dependence on alcohol, tobacco, and caffeine is strongly correlated, the nicotine use of the population was assessed utilizing a five-point scale (where 1 = never vs. 5 = regularly smokes). Around 70% of the participants were smoke-free (mean = 1.79, skewness = 1.555). Out of the remaining 30%, slightly more males fume than females (53.7% vs. 46.3%), which is not in full conformity with the national data (34.1 vs. 6.4) (World Health Organization [WHO], 2018). Caffeine intake was also evaluated on a very similar grading scale. Roughly half of the respondents regularly drink coffee with the male/female ratio being 40.4:60.6, while ⅕ of participants never consume it (mean = 3.56, skewness = 0.553).

### Drinking Habits

**FIGURE 4:**
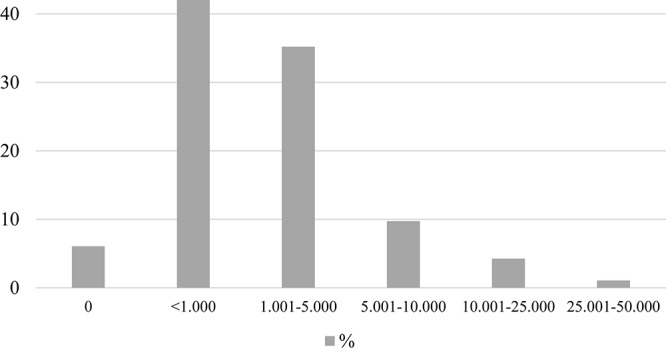
Wine expenditure per month per capita. The self-reported figures are expressed in Hungarian forints (HUF). Current exchange rate: 1 EUR = 325 HUF, 1 USD = 290 HUF, 1 GBP = 360 HUF.

A significantly higher proportion of males drink regularly when compared to females (58.2% vs. 41.8%, respectively). The majority of respondents imbibe wine once or twice a week, but 3.7% enjoy it as many as five times every week. In addition to this, the most popular alcoholic beverages include beer, champagne, and various spirits, which are consumed roughly once a week. While males tend to spend more than females, the self-reported household monthly expenditure on wine is relatively low, typically under 5000 Hungarian forints (€15, $17, £14), and paying over 10,000 forints (€31, $34, £28) per month is infrequent, although there are a few who actually fork out over 50,000 forints/month (€154, $172, £139) for wine. None of the respondents spend in excess of 100,000 forints (or €308, $345, £278) each month on drinks (Figure 4).

This may be related to income level, geographical location, alcohol awareness, and observing drinking guidelines. Nevertheless, it is also pertinent to mention that Hungarian regulations allow families, especially in the countryside, to produce 50L home-made spirits (or equivalent other alcoholic beverages) annually tax-free for domestic consumption only.

### Music and Wine

Wine’s connection to music is elemental: early composers would create symphonies to accompany feasts, and in turn musicians were frequently paid in wine. Ancient records also suggest that new organs would be initiated by having wine poured into their pipes. Supported by recent academic research, music even changes the taste of wine: it not only modifies our wine perception, but actually influences wine-making/characteristics itself as well. In light of this, our aim was to explore the attitude of participants around the relationship between music and wine.

Slightly more than half of the respondents (52.3%) felt that music had no effect on vinification. Over a third (39.7%) assumed that melody improves, a very few believed it deteriorates (1.1%), and some (6.9%) were undecided about its impact on wine quality.

**FIGURE 5:**
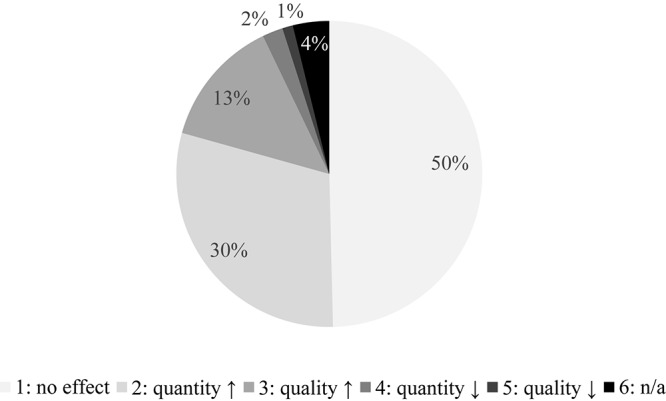
Presumed effect of music on wine consumption. This diagram depicts what participants believe about the role melody plays on wine consumption. (1) There is no effect, or as a result of music, (2) people may drink more, (3) individuals would choose better quality wines, (4) respondents might drink less, or (5) the public could turn to lower-quality products. A small number of participants (3.8%) gave no answer to this question.

Around half (49.6%) of the respondents thought that music had no effect on wine consumption whatsoever. Almost a third (29.7%) felt that it encourages people to drink more, while 13.5% believed it stimulates feasting on higher-quality products. In contrast, only 2.2% expected that music would reduce alcohol intake and 1.1% assumed that it might foster poor quality wine consumption. A few participants (3.8%) gave no answer to this question (Figure 5).

While traditionalism is an inherent characteristic of the wine sector, in an endlessly modernizing world, innovation is inevitable during vinification as well, just as with any other businesses. In fact, consumers tend to try new products; however, their curiosity largely depends on age, personality, income, social status, etc. Fermenting wine from serenaded grapes is certainly a unique approach, and so the disposition of participants toward “music-fed” wines was also evaluated. The majority would definitely try (85.8%) and even buy (68.5%) such alcoholic beverage. The price tag, however, is more divisive among the respondents.

**FIGURE 6:**
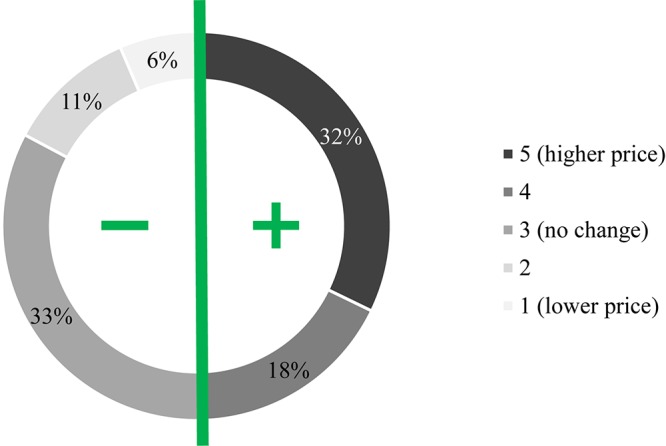
Effect of music as a new attribute on pricing. Participants were asked on a five-point scale whether playing melody to vineyards would eventually influence the price of the end product. While roughly half of the respondents felt that this new attribute should have (1–3) no effect on the goods from a monetary point of view (or such bottles should even be somewhat cheaper), the other half believed music-fed wines must definitely be (4–5) more expensive.

**FIGURE 7:**
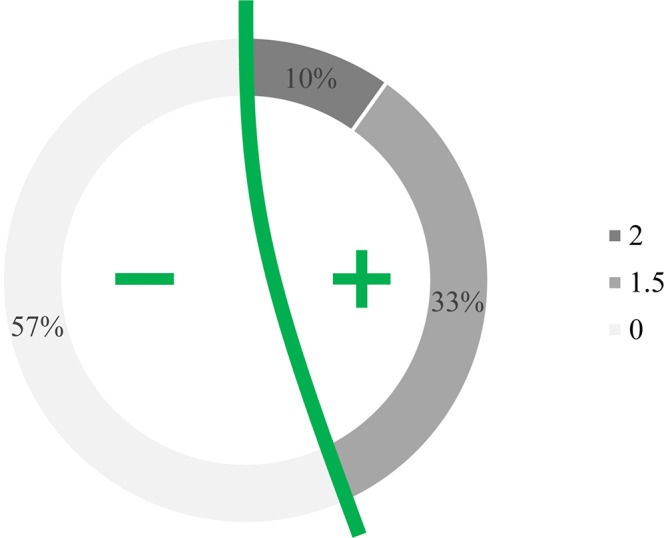
Willingness to pay more for music-fed wines. Graph depicts that while 57% of respondents would not pay more for music-fed wines, 33 and 10% are ready to give 1.5 times more or even twice as much, respectively, than that of the price of any similar albeit not serenaded product.

Because research and development increase production costs, innovation and expensiveness go hand in hand. Consumers are aware of this principle and will build this tenet into their own expectations. However, in light of diverse reservation prices (i.e., the maximum amount an individual is willing to pay for a product), their purchasing potential will also vary. In addition to standard demographic variables (e.g., age, income, etc.), it is mainly influenced by the perceived relevance of the innovation to the customers’ needs when compared to traditional products. Recognizing this, the opinion about pricing a specialty product was also sought in this project using a five-point scale questionnaire, where grades 1–5 denoted zero or negative (i.e., price reduction) or positive (i.e., price increasing) effects of music, respectively. As graphically illustrated in Figure 6, half of the respondents expect no change or a slight price drop, whereas the other half anticipate increased figures (mean = 3.58, skewness = 0.372, mode = 3). In contrast, 43% of the participants are actually prepared to pay more for music-fed wines, although the majority is willing to give only between 1.5 times and twice the normal price for this specialty product (Figure 7).

### Relationship Analysis

**FIGURE 8:**
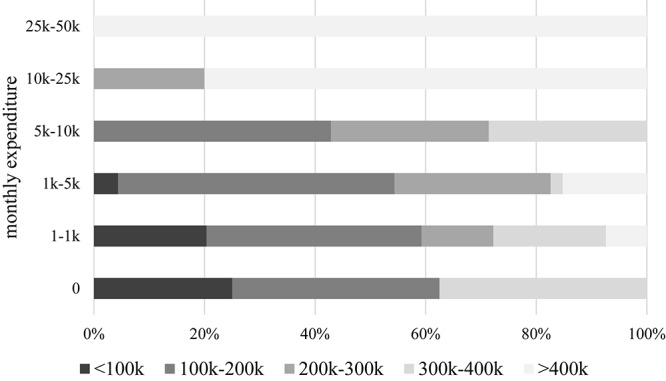
The relationship between the monthly income and the monthly expenditure on wine. The self-reported figures are expressed in Hungarian forints (HUF), where k stands for kilo (i.e., thousand forints). Current exchange rate: 1 EUR = 325 HUF, 1 USD = 290 HUF, 1 GBP = 360 HUF.

**FIGURE 9:**
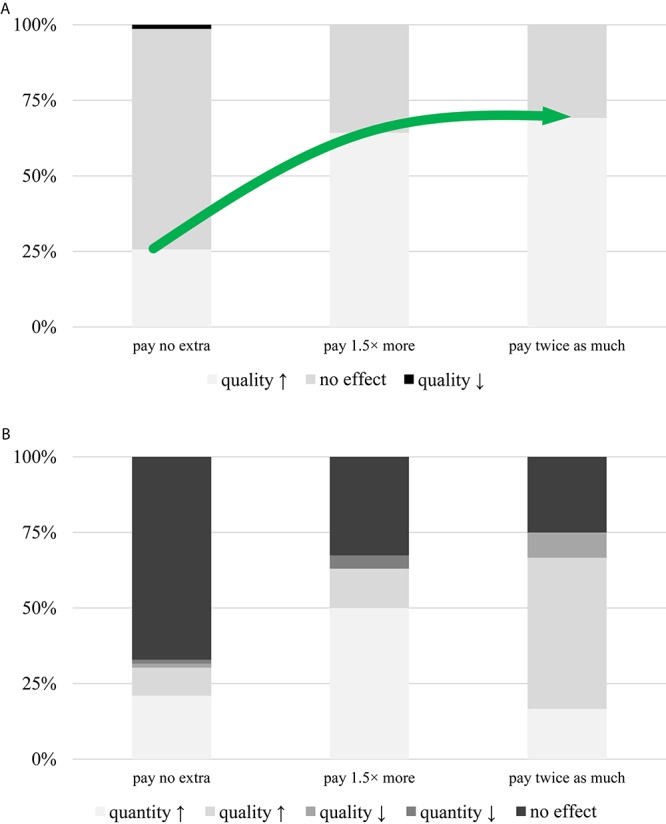
Relationship between belief and willingness to buy. **(A)** Respondents who believe that melody has a beneficial effect on viti-culture are willing to pay more for music-fed wines. **(B)** Participants who feel that it has a positive effect on either quantity or quality of drinking are happy to pay 1.5 times more or even twice as much for this special product, respectively.

**FIGURE 10:**
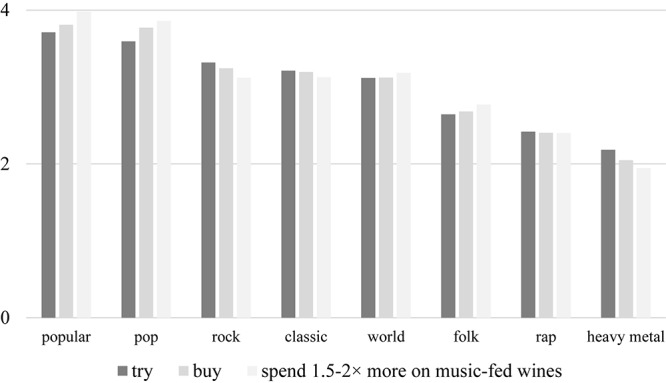
Customer attitude stratified by musical preference. There is only a very weak and non-significant relationship between various customer attitudes (e.g., trying, buying, or spending more, as per the respective five-point grading scales) and the musical style of respondents, the mark that the individual musical preference has no real impact on the participants’ willingness to try, buy, or pay more for music-fed wines.

There is an obvious relationship between the respondents’ self-reported monthly income and their expenditure on wine. Consumers with higher earnings generally buy better quality products. On the other hand, the standard deviation among lower-income individuals is larger, and the relationship is significant and strong (Φ = 0.673, α = 0.000) (Figure 8). After further analysis, it was found that participants who believe in the positive effect of music on vinification are ready to pay more for the special serenaded wines, suggesting that belief is connected to willingness to try/buy. This relationship is significant and moderately strong (Φ = 0.389, α = 0.002) (Figure 9A). In addition, those who believe that music has no effect on alcohol use would not pay more for this specialty product, whereas respondents who are of the opinion that melody encourages people to drink more might pay 1.5 times more for a bottle. On top of that, people who feel that music fosters quality wine consumption would be willing to pay double the price for music-fed wines (Φ = 0.513, α = 0.000) (Figure 9B). However, these observations at hand could be easily confounded by the musical orientation of the individuals, in as much as it is an unverified fact that those who like classical music are not really fond of heavy metal and *vice versa*, but the vineyards were pampered with the masterpieces of Mozart. Regardless of this knowledge, no significant difference was found when data were stratified for musical preference, suggesting that tendencies and personality traits according to their musical genres is largely irrelevant (Figure 10).

**FIGURE 11:**
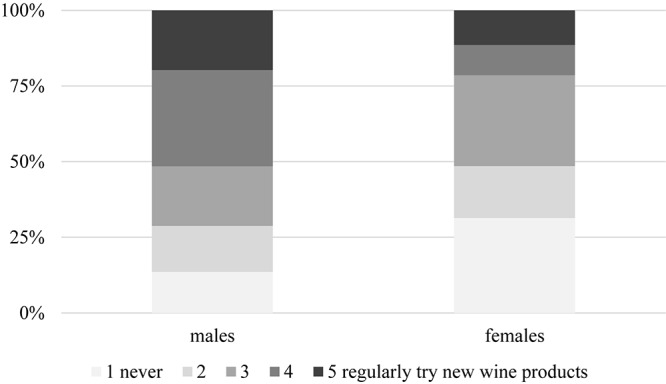
Gender differences in willingness to try new wine products.

**FIGURE 12:**
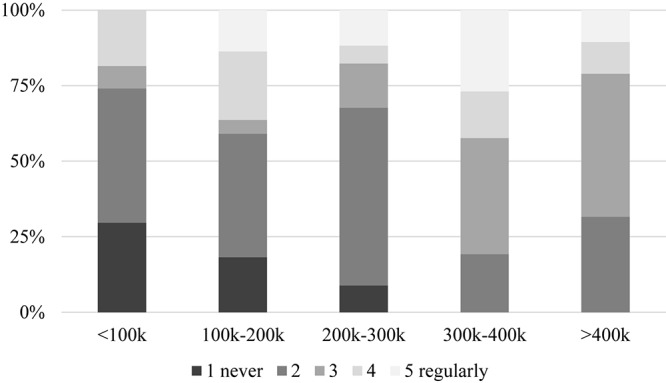
Relationship between income and willingness to try innovative wine products. The self-reported figures are expressed in Hungarian forints (HUF), where k stands for kilo (i.e., thousand forints). Current exchange rate: 1 EUR = 325 HUF, 1 USD = 290 HUF, 1 GBP = 360 HUF.

**FIGURE 13:**
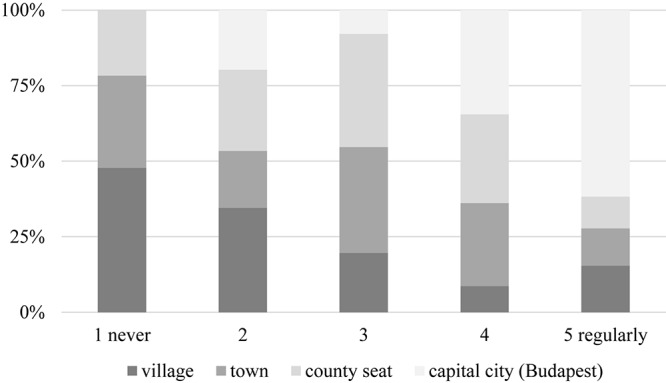
Impact of place of residence on willingness to try unique wine products. As shown in this figure, urban residents are more likely to try new wine products than those living in rural areas.

**FIGURE 14:**
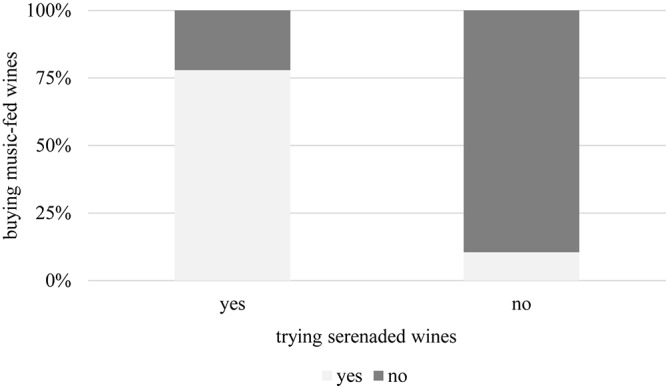
Relationship between trying and buying. Respondents who are more open to try music-fed wines are also significantly more likely to purchase such special product.

There was no substantial correlation between education, marital status, or number of children and consumer behavior. However, a gender difference exists, inasmuch as males in general are more open to try new wine products than females (Φ = 0.339, α = 0.004) (Figure 11). Those with higher income are also more willing to taste unusual alcoholic beverages (Φ = 0.605, α = 0.000, Figure 12), so are urban residents when compared to those living in villages or rural areas (Φ = 0.487, α = 0.001, Figure 13). Individuals who would try music-fed wines are more likely to eventually buy it as well: this relationship is strong and significant (Φ = 0.502, α = 0.000) (Figure 14).

## Discussion

Evaluating how individuals perceive, learn, remember, and are motivated during the buying and consuming process is essential in designing effective marketing strategies. Understanding the brain functions during these events is necessary to influence and persuade potential customers. While there is an obvious difference between the various age and gender groups, each person individually controls the countless information they receive. Neuro-marketing has emerged to assess this selection process, and to shape personalized decision-making.

In this report, we evaluated the disposition of the Hungarian population of unique melody-fed wines. The majority of respondents listen to music regularly and believe that it has a positive impact on the human body. Participants typically drink wine once or twice a week, and their average expenditure on such alcoholic beverage is in the range of 5000 Hungarian forints per month (€15, $17, £14). Roughly third of the population is of the opinion that music prompts consumption of better quality or increased quantity of wine.

Most of the respondents are prepared to try and buy music-fed wines. They also appreciate that innovation comes with a price tag, but this is not directly proportionate to their willingness to actually spend any extra amount on serenaded products: only less than half of the population would pay 1.5–2 times more for that. Household expenditure on alcohol strongly correlates with income; however, a solid belief about the positive effects of melody is needed to spend more on such specialty bottles. While females are largely convinced about the beneficial impact of fine tunes, it is males who drink wine more regularly.

From a neuro-marketing point of view, music-fed wines are best suited for urban male residents on generous payroll. Communicating the positive effects of melody on both grapes and humans as a higher added value is a crucial aspect of targeting such customers and justifying the escalated price of this premium-category product. Connecting sensation-seeking and amusement tourism, e.g., visiting the serenaded plantations, should be a welcome addition among AM-Tokaj’s marketing tools.

## Data Availability Statement

All datasets generated for this study are included in the article.

## Author Contributions

All authors listed have made a substantial, direct and intellectual contribution to the work, and approved it for publication.

## Conflict of Interest

AP is the founding director and owner of AM-Tokaj winery. The remaining authors declare that the research was conducted in the absence of any commercial or financial relationships that could be construed as a potential conflict of interest.

## Footnotes

1 http://www.am-tokaj.wine
